# On the Entropy of Oscillator-Based True Random Number Generators under Ionizing Radiation

**DOI:** 10.3390/e20070513

**Published:** 2018-07-09

**Authors:** Honorio Martin, Pedro Martin-Holgado, Pedro Peris-Lopez, Yolanda Morilla, Luis Entrena

**Affiliations:** 1 Electronic Technology, Carlos III University of Madrid, 28911 Leganés, Spain; 2 Centro Nacional de Aceleradores (CNA), Universidad de Sevilla, CSIC, 41092 Sevilla, Spain; 3 Department of Computer Science, Carlos III University of Madrid, 28911 Leganés, Spain

**Keywords:** TRNG entropy, ionizing radiation, ring oscillator, self-timed ring, NIST

## Abstract

The effects of ionizing radiation on field-programmable gate arrays (FPGAs) have been investigated in depth during the last decades. The impact of these effects is typically evaluated on implementations which have a deterministic behavior. In this article, two well-known true-random number generators (TRNGs) based on sampling jittery signals have been exposed to a Co-60 radiation source as in the standard tests for space conditions. The effects of the accumulated dose on these TRNGs, an in particular, its repercussion over their randomness quality (e.g., entropy or linear complexity), have been evaluated by using two National Institute of Standards and Technology (NIST) statistical test suites. The obtained results clearly show how the degradation of the statistical properties of these TRNGs increases with the accumulated dose. It is also notable that the deterioration of the TRNG (non-deterministic component) appears before that the degradation of the deterministic elements in the FPGA, which compromises the integrated circuit lifetime.

## 1. Introduction

Pico-satellite constellations in Low Earth Orbit (LEO) have become a popular platform that allows earth observation, weather forecasting, space research and communications, among other applications. One of the main advantages of this emerging alternative to a conventional satellite is the cost. Pico-satellites are typically made of Commercial-Off-The-Shelf (COTS) components [1] as Field Programmable Gate Arrays (FPGAs) so the result is an inexpensive satellite network that could be deployed with a minimal budget.

In this framework, the generation of secure encryption keys for use in satellite communications has emerged as a major challenge. The key generation typically relies on True Random Number Generators (TRNG) integrated into pico-satellites [2]. Due to their low-cost nature, TRNGs are embedded in some of the COTS avoiding expensive ad-hoc solutions. Several TRNGs approximations presented in the literature fit this low-cost requirement. Special attention deserves those TRNGs that can be straightforwardly implemented on FPGAs such as [3,4].

Due to their paramount importance in the communication security, these TRNGs are subjected to exhaustive quality randomness analysis on the ground in order to check their suitability for the mission. To that end, several well-known test suites such as NIST [5] or ENT [6] are used to guarantee the entropy of TRNGs in different scenarios including different temperatures, over-clocking, underpowering, etc. [7,8]. In this context, where the TRNGs will be exposed to ionizing radiation from space, it is also necessary to check its functionality under this condition.

The effects of Total Ionizing Dose (TID) in FPGAs have been widely studied in the scientific literature [9,10]. TID effects are typically studied in circuits where for a given particular input, the output is always the same (deterministic logic). Therefore, the effects in this kind of circuits can be quantified using metrics as the Hamming distance between the expected output and the obtained output. A preliminary analysis of the radiation influence on a metastable-based TRNG was reported in [11]. However, TID effects were not studied. In this context, the main contribution of the present research is to study for the first time (to the best of our knowledge) the effects of ionizing radiation on non-deterministic logic as is the case of a TRNG. More specifically, we study the TID effects produced by radiation on two well-known TRNGs [12,13] based on jitter sampling principle. To that end, a FPGA that contains the implementation of the two mentioned TRNGs has been exposed to a Co-60 radiation source. Finally, the statistical properties of the output have been evaluated for different accumulated TID in order to see how radiation affects the randomness quality of the output. The obtained results prove that an unacceptable degradation of the TRNGs happens earlier than the failures in deterministic blocks appear. Thus, the radiation-safe operating range of the entire system will be determined by the TRNGs.

The rest of the paper is organized as follows: Section 2 provides some background on TRNGs, statistical tests and typical effects of TID on Flash-FPGAs. Section 3 describes the experimental set-up. Section 4 presents the experimental results for the accumulated doses. Finally, some conclusions are drawn in Section 5.

## 2. Background

In this section, we provide some background on the related work: True-Random Number Generators, entropy tests and effects of Total Ionizing dose in FPGAs.

### 2.1. TRNGs

TRNGs are used in a wide variety of applications ranging from cryptographic protocols [14] to Monte Carlo simulations [15]. From a security point of view, TRNGs are particularly important because they are typically used to add freshness to cryptographic algorithms. For that reason, the increasing necessity of embedded hardware-based random number sources has spawned a proliferation of embedded TRNGs solutions such as [3,4]. TRNGs that consist of sampling jittery clocks stand out among the different existing proposals for their flexibility and straightforward implementation.

**Figure 1:**
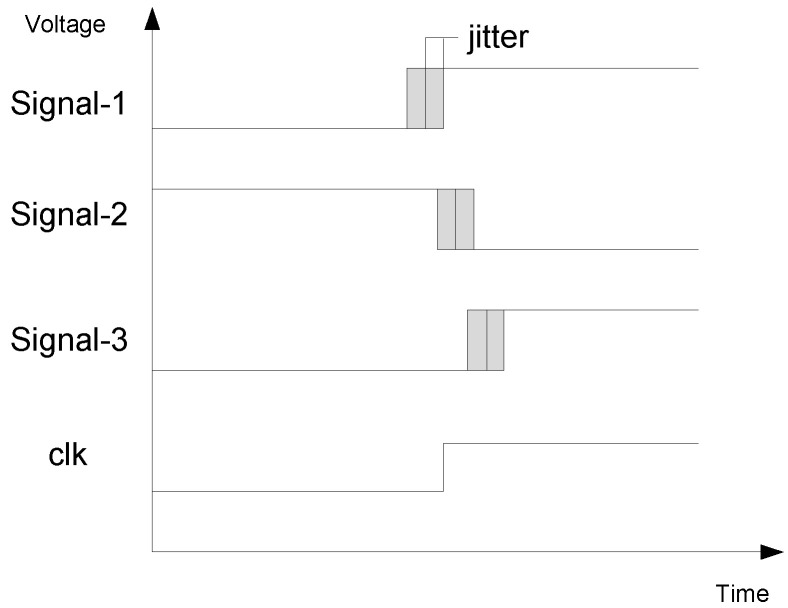
Jitter sampling principle.

Jitter can be defined as the deviation from true periodicity of a presumably periodic signal. This deviation can come from deterministic sources or random jitter sources as thermal noise. The entropy extraction principle of this kind of generators is depicted in Figure 1. Several high-frequency oscillators are sampled by a reference clock. If at least one of these oscillators is sampled in the jitter zone, the addition of all the samples will be random. A higher number of oscillators will increase the possibilities of sampling at least one in the jitter zone.

**Figure 2:**
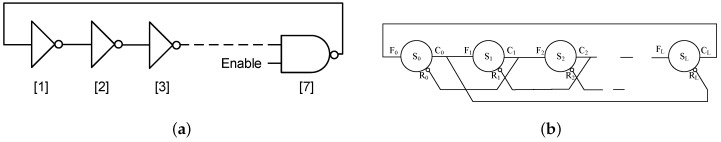
High-frequency oscillators. (**a**) RO scheme with enable. (**b**) Self-Timed Ring structure.

In this work, we have selected two representative TRNGs based on jitter sampling that use two different kinds of high-frequency oscillators. On the one hand, Wold et al. proposal [12] uses Ring Oscillators (ROs) as high-frequency clocks. A RO is a circuit composed of an odd number of inverters in a ring, whose output oscillates. In Figure 2a a typical RO scheme is depicted. On the other hand, the Cherkaoui TRNG [13] generates the high-frequency signals using a Self-Timed-Ring (STR). An STR is a n-stage micropipelined architecture that implements a handshake protocol that guarantees the distribution of events through the different stages. Each stage consists of a Muller gate and an inverter. Figure 2b presents a STR architecture. We urge the reader to consult the original works [12,13], for further details about the implementation specifications and stochastic models linked to each TRNG.

### 2.2. Entropy Tests

TRNGs are typically subject to exhaustive tests due to their critical role in security systems. There are currently three trends which can be used to evaluate the quality of a TRNG:

- **Output Statistical Analysis:** the most extended way of assessing the TRNG quality is testing the statistical distribution of the output using statistical tests [16]. Traditionally, widely known test suites as NIST or Diehard have been used to obtain an initial evaluation of TRNGs [5,6]. These tests cannot guarantee the entropy of the TRNG because they check the final output (after the post-processing) of the TRNG.
- **Entropy Source Statistical Analysis:** a new trend in TRNG evaluation was introduced in AIS-31 [17] where not only the final TRNG output is evaluated but also the entropy source. Among the different testing approaches stand out the NIST recommendations about entropy sources that include some statistical tests intended for estimating the min-entropy of a random number generator [18]. Of particular interest are the tests intended for generators that may have dependencies in time and/or state, which are commonly known as non independent and identically distributed (non-IID) number generators. These tests are particularly designed to avoid an overestimation of the entropy of these generators.
- **Physical Parameters Analysis:** the estimation of entropy must be based on a carefully constructed model of the random number generation process. Once the stochastic model is set, the measurement of some physical parameters (e.g., jitter measurement) can be used to estimate entropy at the output of the generator. In this line, some interesting proposals have been presented [19,20].

### 2.3. TID on Flash-Based FPGAs

Prolonged exposure of electronics devices to ionizing radiation (particle radiation and high-energy electromagnetic radiation) can cause cumulative effects known as total ionizing dose effects. The ionization dose is deposited by particles passing through the materials changing their electrical properties. From the TID point of view, FPGAs are very complex circuits because they not only include programmable logic and memory to implement designs, but also several peripheral blocks intended for programming and testing functions.

In flash-based FPGAs, the TID effects at device level are focused on the floating gate and CMOS transistors. The floating gate can be affected by three different radiation-induced phenomena which reduce its threshold voltage: holes injected into the floating gate, holes trapped into the oxides and electrons emitted over the polysilicon/oxide barriers [9]. Regarding the CMOS transistors, it is necessary to distinguish between NMOS and PMOS transistors. For NMOS, trapped holes tend to decrease the threshold while interface states tend to increase it. On the other hand, in PMOS transistors, trapped holes and the interface states tend to increase (in absolute value) the threshold voltage [10]. In low voltage devices with thin oxide, TID effects in CMOS transistors are negligible.

All in all, these radiation-induced deteriorations of the electrical characteristics will result in a degradation of the propagation delay and an increase of the core power supply current and threshold voltage ($V_{t}$) of the FPGA switches.

## 3. Experimental Section

In this section, we present the different elements that will be involved in our experimental setup.

### 3.1. TRNG Implementations and Tests

In this work, the selected commercial FPGA is an Igloo AGLN250, manufactured by Microsemi. This FPGA is based on a 130 nm flash technology. The clock reference is the on-board 20 MHz crystal oscillator. A high precision voltage source is used to set the voltage operation in order to avoid influences in the randomness due to the power supply.

On the one hand, a RO-TRNG scheme composed of 512 identical laid-out ROs has been implemented as described in [12]. Each RO consists of a chain of 5 inverters. On the other hand, a STR-TRNG consisting of 511 stages has been implemented following the design introduced in [13]. The STR has been properly configured in order to guarantee the correct distribution of events. The TRNGs have been placed in different FPGA zones in order to avoid mutual influences.

The TRNG outputs have been collected and transferred to a host computer using an RS232 communication protocol. The integrity of the transmitted information has been ensured using a CRC mechanism. The achieved throughput for both TRNGs is 25 Mbps because all oscillators (ROs and STRs) are sampled using the reference clock. Regarding the used resources of the FPGA provided by the synthesis tool, a total of 2976 Core tiles (1997 Combinational tiles and 979 Sequential tiles), occupying a 16.67% of the available resources in the FPGA, are used.

Once the data is on the host computer, two NIST test batteries have been used to evaluate the TRNG quality [5,18]. On the one hand, we have checked the final TRNG output using the well-known NIST test suite that includes several statistical tests as the frequency test, runs, binary matrix rank, etc. On the other hand, we have estimated the min-entropy by using some tests recommend in [18] for testing non-IID generators. We have discarded the measurement of a physical parameter (in this case the jitter) because of the complexities of the radiation set-up.

### 3.2. TID Setup

The main source in accordance with the standard test method [21] to perform Total Ionizing Dose tests in space industry is the electromagnetic radiation (photons) from Co-60. For this experiment it was provided by the Gamma Radiation Laboratory (RadLab) of the Centro Nacional de Aceleradores (CNA), in Seville. This is a joint center of the Universidad de Sevilla (Spain), Junta de Andalucía and CSIC.

The Co-60 radioactive source available at CNA is installed in a Gammabeam X200 irradiator from Best Theratronics [22,23]. The photon energies are 1.17 MeV and 1.33 MeV.

**Figure 3:**
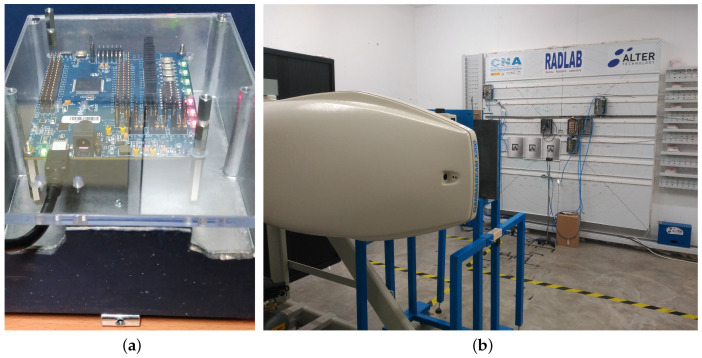
Radiation setup. (**a**) DUT setup; (**b**) Irradiator.

The DUT and the PCB where it is soldered were placed into a filter box of 12 cm × 17 cm to be submitted to radiation, according to [21]. This container has 2 mm of aluminum and 1.5 mm of lead in the outer layer, and a front cover of 5 mm of PMMA (poly-methilmetacrilate) to achieve the charged-particle equilibrium. The distance between the filter box and the Co-60 was 302 cm. Figure 3 shows the DUT inside the filter box and its placement in the radiation facility.

The dose rate was measured in the four corners of the filter box (inside) using two Farmer ionization chambers connected to a MULTIDOS electrometer, all of them by PTW. First air kerma rate was obtained, then the dose rate in silicon (Si) was finally calculated considering the conversion factors. The dose rate uniformity calculated from the four dose rates previously mentioned was 98.2% (a minimum of 90% is required by [21]), and the corresponding average dose rate was 217.0 rad(Si)/h. The total accumulated dose of the test was 45 krad(Si).

## 4. Experimental Results

**Figure 4:**
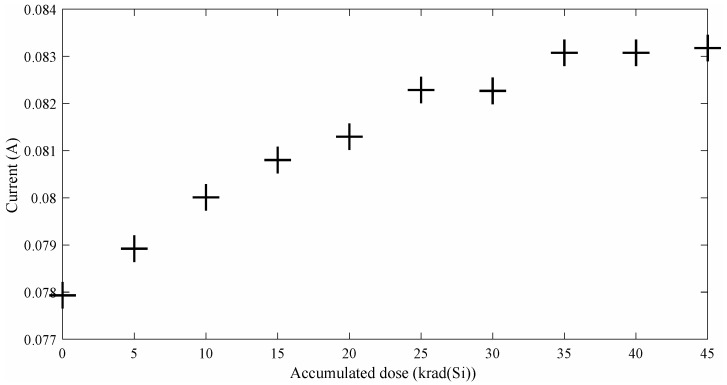
FPGA current (A) vs Total Ionizing Dose [krad(Si)].

As stated before, the FPGA power supply has been continuously monitored during the experiment. In Figure 4 is depicted the FPGA current during the experiment. As reported in other similar FPGA radiation experiments [24], the current increases almost linearly with the accumulated dose. It is important to note that during all the experiment the current is within the limits of the manufacturer recommendations for this board. At pre-irradiation conditions, the measured current was 77.9 mA, reaching 83.2 mA at the end of the experiment [45 krad(Si)]. Meanwhile, the power supply was fixed to 5 V. The first communication error appeared at 38.1 krad(Si) (83.1 mA) and the final valid bit-stream (correct CRC) was captured at 40.8 krad(Si) (83.2 mA). These results are in line with those reported in [24] where some Igloo FPGAs were irradiated at room temperature using JPL’s Co-60 source. In these experiments, functional failures appear from 30 krad(Si) to 40 krad(Si) of accumulated dose.

Finally, in Table 1 the *p*-values obtained with NIST tests suite are summarized for the two TRNGs at pre-irradiation conditions.

### 4.1. TRNG-RO Experimental Results

**Figure 5:**
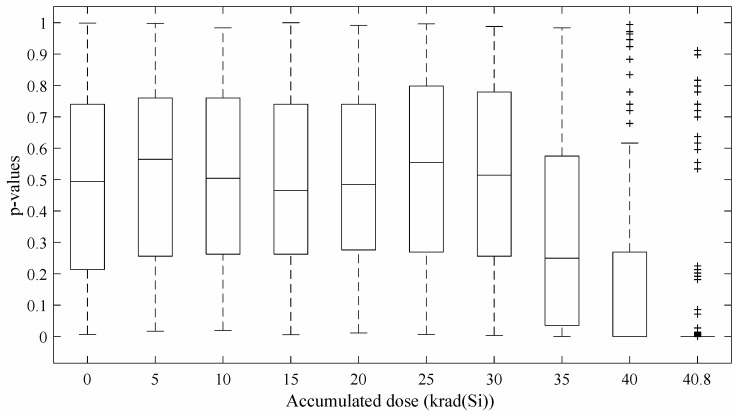
Wold et al. *p*-value distributions for different accumulated doses.

**Figure 6:**
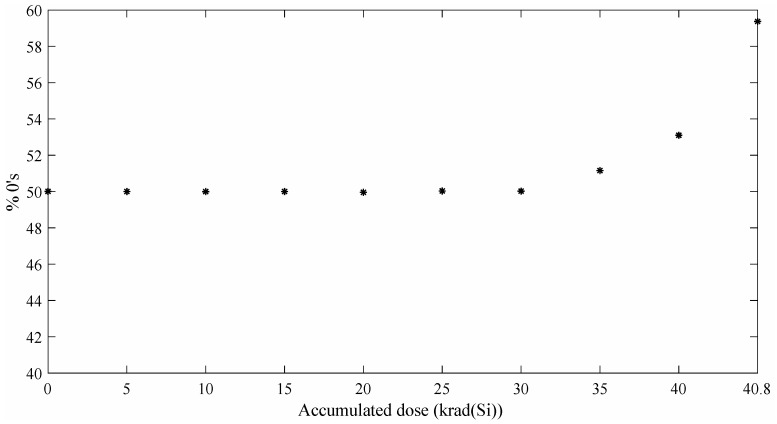
Wold et al. % 0’s for different accumulated doses.

The bit-stream quality of the Wold et al.’s TRNG [12] has been checked using two well-established NIST statistical test suites [5,18]. To that end, 5 Mbit of data were collected for each accumulated dose. Figure 5 shows boxplots of the *p*-value distribution (NIST tests) for different accumulated doses. It can be appreciated that the quality of the output decreases with the accumulated dose. Please note that a random generator passes the NIST tests whether the obtained *p*-values are uniformly distributed on the interval [0,1]. In our particular case, NIST tests are passed until 35 krad(Si) are reached. After this point, the quality of the TRNG output degrades rapidly. This phenomenon can be explained if we pay attention to the ratio of 0’s/1’s generated by the TRNG. In Figure 6 are depicted the percentages of 0’s of each bit-stream. It can be seen that for an accumulated dose lower than 35 krad(Si), the distribution is almost ideal (50%). From this point to the end, the number of 0’s in the bit-stream increases reaching a 59.3%. This bias can be explained because a lower oscillation frequency of the RO means that there are fewer events to sample in the jitter zone producing a higher number of deterministic bits. The lower oscillation frequency is induced by the accumulated dose as explained in Section 2.

Regarding the min-entropy of the entropy source, Table 2 summarizes the entropy per bit estimation of the tests included in SP 800-90B Entropy Estimation Suite [18]. The same trend than before (the degradation of the randomness quality is noticeable from 35 krad(Si)) can be appreciated due to the direct relation between the quality of the entropy source and the quality of the output. It is worth noting that the estimated min-entropy is set to the lowest value of the ten estimation methods computed. In our particular case, collision, t-Tuple and LSR tests determine the min-entropy value. The first one [25] gives a measure of the mean number of samples to the first collision in a dataset, where a collision is any repeated value. The t-Tuple estimation examines the frequency of pairs, triples, etc. in the bit-stream and produces an estimate of the entropy per sample based on these frequencies [18]. Finally, LSR checks the IID assumption using the length of the longest repeated substring. As the number of 0’s increases with the total ionizing dose, it will be more likely to have a collision or a higher frequency of pairs and triples, and as consequence of this the min-entropy will be lower.

### 4.2. TRNG-STR Experimental Results

**Figure 7:**
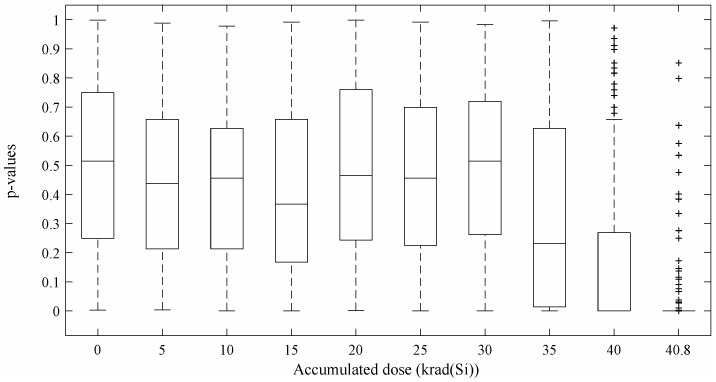
Cherkaoui et al. *p*-value distributions for different accumulated doses.

**Figure 8:**
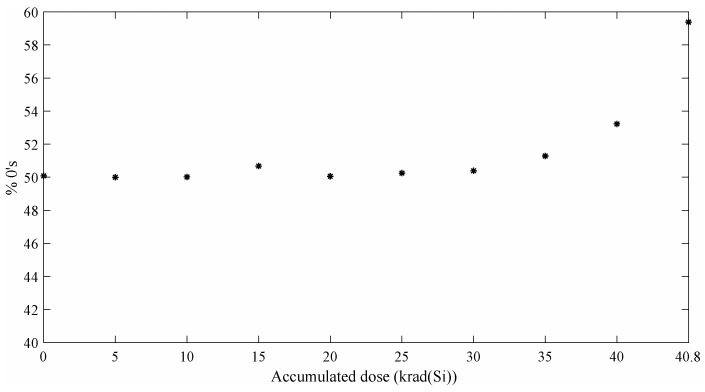
Cherkaoui et al. % 0’s for different accumulated doses.

For the Cherkaoui et al. TRNG [13], the analysis of 5-Mbit bit-streams for each accumulated dose has derived in the *p*-value distribution showing in Figure 7 for the NIST statistical test suite. The results obtained for this TRNG are very similar to those reported for the Wold et al.’s TRNG [12]. Once again, the TRNG passes all the test until 35 krad(Si) are reached. From this point, a rapid degradation on the output quality can be observed. The same bias than in the RO-TRNG case appears in the bit-stream (Figure 8). In this case, the percentage of 0’s in the bit-stream reaches the 59.4% for an accumulated dose of 40.8 krad(Si).

The analysis of the entropy source as a non-IID source is shown in Table 3. Positive results have been obtained until an accumulated dose of 35 krad(Si). Once again, the min-entropy is set by two estimators (Collision and t-Tuples) because of the bias that appears in the bit-stream.

All in all, the results obtained for both TRNGs show that before the first failure of a deterministic block of the design (38.1 krad(Si)), a degradation on the statistical properties of both TRNGs is produced. This degradation can compromise the security of applications that depend on the freshness generated by TRNGs. This outcome highlights the necessity of exhaustive testing of TRNGs under ionizing radiation. In addition, the accumulated dose that these devices can support might be revisited where implementing this kind of TRNGs.

## 5. Conclusions

In this work, we have addressed the random number generation issue under conditions of ionizing radiation. This condition can be found in LEO where many pico-satellites that use FPGAs among other components are deployed. The functionality of deterministic logic implemented on FPGAs under ionizing radiation has been investigated deeply by the scientific community. Nevertheless, up-to date, the influence of ionization over non-deterministic components such as cryptographic components has not been as well studied. In this vein, we have tested the influence of ionizing radiation over two well-known TRNGs based on sampling jittery signals [12,13]. We have used two different NIST test suites [5,18] to evaluate the randomness quality (e.g., entropy or linear complexity) of both TRNGs for different accumulated doses. During the experiment, a degradation of the statistical properties of both TRNGs was observed. It is very remarkable that deterioration occurs before the first failure in the deterministic blocks. Therefore, the non-deterministic component (TRNG in our particular case) determines the maximum level of radiation allowed in the integrated circuit. All this points out the importance about considering the non-deterministic components when radiation is at stake.

## Figures and Tables

**Table 1:** NIST STS Results.

Test	RO-TRNG	STR-TRNG
Frequency	0.911413	0.253551
Block Frequency	0.804337	0.082177
Cumulative Sums	0.476471	0.215914
Runs	0.671779	0.804337
Longest Run	0.949602	0.991468
Rank	0.253551	0.862344
FFT	0.148094	0.739918
Non-Overlapping Template	0.462714	0.479021
Overlapping Template	0.534146	0.299251
Universal	0.253551	0.299251
Approximate Entropy	0.066882	0.082177
Random Excursions	0.633125	0.257812
Random Excursions Variant	0.508011	0.135850
Serial	0.789259	0.504774
Linear Complexity	0.213309	0.122325

**Table 2:** NIST min-entropy RO Results.

	0 krad(Si)	5 krad(Si)	10 krad(Si)	15 krad(Si)	20 krad(Si)	25 krad(Si)	30 krad(Si)	35 krad(Si)	40 krad(Si)	40.8 krad(Si)
**Most Common Value**	0.99861	0.998628	0.998306	0.998273	0.997432	0.998117	0.998272	0.965788	0.912059	0.75138
**Collision**	0.944718	0.944718	0.928538	0.944718	0.928538	0.955606	0.955606	0.785681	0.682587	0.447331
**Markov**	0.998805	0.99911	0.998105	0.998776	0.997169	0.998986	0.998619	0.933978	0.841065	0.605037
**Compression**	1	1	0.970713	1	0.97486	1	1	0.819469	0.723149	0.527182
**t-Tuple**	0.933664	0.931583	0.935803	0.935803	0.931583	0.933664	0.933664	0.90872	0.866229	0.60287
**LRS**	0.98132	0.973757	1	0.999913	0.999368	0.981764	0.912897	0.991443	0.932321	0.832981
**MultiMCW Prediction**	0.999113	0.998501	0.999401	0.998223	0.998953	0.999325	0.999181	0.971087	0.912829	0.736576
**Lag Prediction**	0.998727	0.99869	0.998496	0.998613	0.997539	0.999286	0.999181	0.963841	0.912324	0.751094
**MultiMMC Prediction**	0.998784	0.999426	0.998903	0.998675	0.998834	0.998969	0.998536	0.962938	0.882302	0.586627
**LZ78Y Prediction**	0.99864	0.99906	0.998615	0.998359	0.997974	0.999135	0.998135	0.96379	0.912679	0.751365
**min-entropy**	**0.933664**	**0.931583**	**0.928538**	**0.935803**	**0.928538**	**0.933664**	**0.912897**	**0.785681**	**0.682587**	**0.447331**

**Table 3:** NIST min-entropy STR Results.

	0 krad(Si)	5 krad(Si)	10 krad(Si)	15 krad(Si)	20 krad(Si)	25 krad(Si)	30 krad(Si)	35 krad(Si)	40 krad(Si)	40.8 krad(Si)
**Most Common Value**	0.996126	0.997298	0.99782	0.979399	0.997371	0.990443	0.987387	0.962743	0.908064	0.750457
**Collision**	0.944718	0.928538	0.939304	1	0.933911	0.944718	0.944718	0.782042	0.682587	0.446371
**Markov**	0.996467	0.996604	0.99818	0.982565	0.997971	0.990434	0.987576	0.930175	0.83738	0.604465
**Compression**	0.931584	0.933664	0.927586	0.933664	0.935803	0.931584	0.935803	0.856409	0.863284	0.629717
**t-Tuple**	0.931584	0.933664	0.927586	0.933664	0.935803	0.931584	0.935803	0.856409	0.863284	0.629717
**LRS**	0.952589	0.994943	0.932321	0.99972	0.996622	0.998976	0.999929	0.987735	0.932321	0.839317
**MultiMCW Prediction**	0.998189	0.998657	0.998235	0.987077	0.999218	0.996626	0.996279	0.962677	0.908805	0.680523
**Lag Prediction**	0.975427	0.998682	0.997838	0.997586	0.999067	0.997723	0.998898	0.962677	0.911805	0.69826
**MultiMMC Prediction**	0.996487	0.998412	0.998411	0.979472	0.998083	0.990706	0.987457	0.96084	0.880258	0.586456
**LZ78Y Prediction**	0.996362	0.997591	0.998346	0.97946	0.997859	0.990483	0.987461	0.962788	0.908079	0.680523
**min-entropy**	**0.931584**	**0.928538**	**0.927586**	**0.933664**	**0.933911**	**0.931584**	**0.935803**	**0.782042**	**0.682587**	**0.446371**
